# Prevalence of hepatitis B virus infection among pregnant women in Africa: A systematic review and meta-analysis

**DOI:** 10.1371/journal.pone.0305838

**Published:** 2024-07-16

**Authors:** Yilma Markos Larebo, Abebe Alemu Anshebo, Ritbano Ahmed Abdo, Sujit Kumar Behera, Natarajan Gopalan

**Affiliations:** 1 Department of Epidemiology and Public Health, School of Life Science, Central University of Tamil Nadu, Thiruvarur, India; 2 Department of Epidemiology, School of Public Health, Wachemo University, Hossana, Ethiopia; 3 Department of Midwifery, School of Nursing, Wachemo University, Hossana, Ethiopia; Kwame Nkrumah University of Science and Technology, GHANA

## Abstract

**Introduction:**

Africa exhibits a considerably high prevalence of the hepatitis B virus among pregnant women. Furthermore, there is a discernible lack of a well-established surveillance system to adequately monitor and comprehend the epidemiology of the hepatitis B virus, particularly among pregnant women. The eradication efforts of the virus in Africa have been impeded by the significant disease burden in the region, and there is a lack of evidence regarding the pooled prevalence of the hepatitis B virus in Africa. Consequently, this systematic review and meta-analysis aims to determine the prevalence of hepatitis B virus infection among pregnant women in Africa.

**Methods:**

We conducted a systematic literature search using reputable databases such as PubMed, Advanced Google Scholar, Scopus, and the Cochrane Library. The search spanned from July 2013 to July 2023 and included all relevant articles published within this period. To identify potentially eligible articles, we conducted a comprehensive manual review of the reference lists of the identified studies. Our review encompassed articles from the African Journal Online. The analysis focused on observational studies published in peer-reviewed journals that reported the prevalence of hepatitis B surface antigen-positive testing among pregnant women. We utilized the Newcastle-Ottawa critical appraisal checklist to assess the methodological quality of each paper. Finally, a meta-analysis was conducted using a random-effects model.

**Results:**

Out of the 774 studies identified, 31 studies involving 33,967 pregnant women were selected for the meta-analysis. According to the random-effects model, the combined prevalence of hepatitis B virus among pregnant women was 6.77% [95% CI: 5.72, 7.83]. The I^2^ statistic was calculated to be 95.57% (p = 0.00), indicating significant heterogeneity among the studies. The high I^2^ value of 95.57% suggests a substantial degree of heterogeneity. A subgroup meta-analysis revealed that factors such as time-dependent bias, sample size dependence, or individual variation among study participants contributed to this heterogeneity (p-difference < 0.05).

**Conclusion:**

According to the findings of this study, the pooled prevalence of hepatitis B infection among pregnant women in Africa was found to be intermediate-high. It is recommended that policymakers implement hepatitis B virus immunization programs targeting pregnant women and their new-born babies at higher risk of exposure.

## 1. Introduction

Hepatitis is a medical condition characterized by liver inflammation [1–3]. Various virus families are known to cause liver damage [1, 4, 5]. Several medically necessary viruses pose a significant risk to the well-being of millions of people worldwide [3, 4, 6]. Hepatitis B virus (HBV) is a substantial global public health issue [7–9] and belongs to the hepadnavirus family, a group of deoxyribonucleic acid (DNA) viruses [2]. It primarily affects the liver and can lead to both short-term and long-term health problems [8, 9], resulting in significant levels of illness and death [7, 10–14]. World Health Organization (WHO) estimates that 254 million people will be living with chronic hepatitis B infection in 2022 [15]. It is estimated that over 1 million individuals die each year due to chronic liver disease caused by this virus [10, 12].

The hepatitis B virus is a significant public health concern and is particularly widespread among pregnant women in sub-Saharan Africa [16, 17]. It is estimated that approximately 65 million individuals in Africa are infected with HBV, with a mortality rate of 25% [8]. Among pregnant women, the prevalence of HBV infection in different countries of the African region was reported as 7.5% in Sudan [18], 9.3% in Kenya [19], 3.2% in Eritrea [20], and 3.1% in Rwanda [21]. The prevalence of HBV among pregnant women in sub-Saharan Africa ranges from 9–20% [8], posing a persistent and significant public health challenge, particularly in this region. The infection is associated with severe complications, including cirrhosis and liver cancer [8, 16, 17, 22], can be transmitted prenatally, through unprotected sexual contact, intravenous drug use, contaminated blood, and blood products, contaminated injections during medical procedures, and injection drug use and unsafe medical practices [10, 23].

Hepatitis B virus infection demonstrates a noteworthy inclination for vertical and horizontal transmission, posing potential risks to mothers and infants [16, 17, 24]. Consequently, adverse birth outcomes, including stillbirth, fetal loss, neonatal death, premature birth, and low birth weight, may ensue [23]. The risk of acquiring chronic HBV infection varies with age. Around 90% of infections occur during the perinatal period [11]. If an infant’s mother is positive for both hepatitis B surface antigen (HBsAg)and hepatitis B e antigen (HBeAg), there is a 90% likelihood of the infant developing chronic infection by age 6 [12]. For children above six years old, the chances of developing chronic hepatitis B virus are 30–50% and 5–10% [25]. Pregnant women who test positive for both HBsAg and HBeAg have a 70–90% chance of transmitting the infection to their new-born infants. If they test positive for only HBsAg, the chances decrease to 10–40% [8].

In order to prevent up to 96% of transmission, pregnant women should be routinely evaluated for HBsAg, and new-borns whose mothers test positive should receive the HBV vaccine at delivery [8, 26]. The majority of pregnant women, however, do not receive routine screening during antenatal care (ANC) [27]. For both the mother and the new-born, an HBV-complicated pregnancy causes challenges in the management of drugs or medication administration [14]. Despite a potent vaccination, HBV infection remains one of the significant public health issues, primarily in developing nations [28].

The global hepatitis strategy of the WHO, which is endorsed by all member states, aims to reduce new hepatitis infections by 90%, decrease deaths by 65%, and provide treatment for 80% of those living with these illnesses between 2016 and 2030 [29]. Given the scope and severity of the issue, prevention and control of viral hepatitis require a high level of attention [3]. Therefore, controlling the HBV among pregnant women may help break the chain of transmission [7]. However, global progress toward the elimination goals has been slow, particularly in Africa, where the burden of HBV is high [27, 30] and with also a poor uptake of HBV vaccination and an incomplete HBV three-dose vaccine coverage, HBsAg prevalence in the WHO Africa region in remains high [31].

Furthermore, there is a significant deficiency in an efficient surveillance system for monitoring and understanding the epidemiology of HBV among pregnant women [32]. Despite the existence of several studies investigating the prevalence of HBV and its related factors in Africa, the outcomes of this study will provide valuable insights for the development of appropriate strategies to mitigate HBV infection among pregnant women. Currently, there is a lack of comprehensive studies with a specific focus on pregnant women in Africa. This systematic review and meta-analysis aims to determine the pooled prevalence of hepatitis B virus infection among pregnant women. This study’s findings can inform the development of surveillance strategies, advocate for early screening during pregnancy, and facilitate the formulation of effective preventive measures. Hence, the objective of this meta-analysis is to determine the prevalence of hepatitis B virus infection among pregnant women in Africa.

## 2. Methods

### 2.1. Protocol for the study and reporting

The Preferred Reporting Items for Systematic Reviews and Meta-Analyses (PRISMA) standards for the literature search approach, study selection, data extraction, and outcome reporting were followed when conducting this systematic review and meta-analysis (SRMA). The Newcastle-Ottawa scale (NOS) review guideline was modified to create eligibility criteria based on the Condition, Context, and Population (COCOPO) principle [33]. Zotero (version 6.0) reference management software and Rayyan software tools were used to download, organize, review, and cite related articles in systematic and meta-analysis literature reviews [34]. The protocol has been identified as CRD42023442426 and registered in the PROSPERO database.

### 2.2. Variables and measures

These systematic reviews and meta-analyses encompassed all observational research studies conducted in Africa that examined the prevalence of HBV infection among pregnant women. This includes cohort, case-control, and cross-sectional studies that assessed the presence of HBV through the detection of positive hepatitis B surface antigen (HBsAg) using rapid diagnostic tests (RDTs), enzyme-linked immunosorbent assay(ELISA), or a combination of both.

### 2.3. Inclusion and exclusion criteria

We included a comprehensive range of observational studies, such as cross-sectional, case-control, prospective, and retrospective cohorts, that reported their findings in peer-reviewed journals. Moreover, we considered studies eligible for inclusion if they were conducted in Africa, focused on screening pregnant women for HBV, and published in English. Furthermore, our study exclusively incorporates research that adheres to rigorous methodological standards determined by the NOS [33]. We excluded studies, including case reports and case series, from our analysis. For studies published in multiple reports, we excluded duplicates and those that did not involve pregnant women. Additionally, we did not consider studies published in questionable, scholarly, open-access (predatory) journals, following the guidelines provided by Ross-White and colleagues [35].

### 2.4. Study design and search strategy

We conducted an extensive literature search to locate previous systematic reviews, meta-analysis studies, and protocols relevant to the topic of interest. This search used the PROSPERO database and the abstracts of effects (DARE) reviews to ensure no similar study had been registered and published. Various biomedical databases, including PubMed, Advanced Google Scholar, Scopus, and the Cochrane Library, were utilized to search for eligible articles published between July 18, 2013, and July 18, 2023. We expressly limited our search to studies published in English. To identify potentially eligible articles, we thoroughly reviewed the reference lists of the identified studies, which also included articles from the African Journal Online (AJO). To refine our search strategy, we combined medical subject headings (MeSH terms), keywords, and Boolean operators (AND and OR). Additionally, we conducted a secondary search using all identified keywords and index terms. Finally, we examined the reference lists of identified papers and articles to identify any further relevant studies. The database search was performed using terms such as "Prevalence," "Hepatitis B Virus," "Infection," "Pregnant Women," and "Africa" (S1 File).

### 2.5. Study selection and screening

The study selection process was conducted in two stages, following strict inclusion and exclusion criteria. Initially, two authors (YML and AAA) independently evaluated the titles and abstracts of the articles. Subsequently, the same two authors (YML and AAA) individually obtained and scrutinized the complete papers of the eligible articles. To ensure consensus, all three authors (SBK, NG, and RAA) agreed upon the selected articles at each stage of the screening process. In case of any disagreements, a third author (NG) was consulted to resolve them.

### 2.6. Data extraction

The authors’ pre-tested technique was used to extract the data from the included studies. The first author’s last name, the publication year, the study location, the study design, the study year, the HBV-specific antigen reported, the screening method, the number of pregnant women who were screened for HBV (HBsAg), the number of pregnant women who were screened but tested positive for HBV (HBsAg+), how many HBV-infected women tested positive for HBeAg, as well as the risk variables that have been publicly disclosed were all retrieved by two authors (YML and AAA). For further information, the authors of the included studies were contacted when needed. Three additional writers (NG, SBK, and RAA) randomly chose and cross-checked the extracted data.

### 2.7. Measures for data quality control

The methodological quality of each publication in cross-sectional and cohort studies was assessed using the NOS critical evaluation checklist [33]. This measure was also used to determine the likelihood of biased study results. The NOS employs a star rating methodology to assess the risk of bias in three areas: study group selection, group comparability, and result determination. The NOS awards a maximum of ten stars for each study. Based on the NOS classification for quality evaluation in cross-sectional studies, articles were categorized as unsatisfactory (0–4 stars), satisfactory (5–6 stars), good (7–8 stars), or very good (9–10 stars) [36]. Conversely, in cohort studies, acceptable quality included good (1–2 stars in comparability, 3–4 stars in selection, and 2–3 stars in result or exposure domains), fair (1–2 stars in comparison, 2–3 stars in outcome, and two stars in selection domains), and poor (0–1 star in selection, 0 stars in comparability, or 0–1 star in outcome or exposure domains) [33]. Only studies that scored at least seven out of ten were included in our review study after being evaluated against these criteria. Two authors (Y.M.L. and A.A.A.) independently assessed the quality of each study, and any discrepancies were resolved through discussion with a third independent reviewer (NG) (S2 File).

Where: *(asterisks) correspond to ratings assigned for each item according to the Newcastle Ottawa Quality Assessment Scale; HbcAb: hepatitis B core antibody; IGM: Immunoglobulin M; HBV: hepatitis B virus; HDV: hepatitis D virus; ANC: antenatal care; ELISA: Enzyme-linked immunosorbent assay; PCR: polymerase chain reaction; HBsAg: hepatitis B surface antigen; HBs: anti-Hepatitis B surface; HBe: anti-Hepatitis B envelope; HBc: anti-Hepatitis B core; HBeAg: hepatitis B envelope antigen; HBcAg: hepatitis B core antigen.

### 2.8. Statistical analysis

The statistical software package Stata (version 16.0, Stata Corp. LP based in College Station, United States of America) was used to conduct all analyses. A random effects meta-analysis model was used. This model was based on the DerSimonian, and Laird approaches and was employed to combine the prevalence of hepatitis B virus in pregnant women in Africa. Statistical significance was determined using a p-value and a 95% confidence interval. The random effect model was used for analyses with statistical heterogeneity, while the fixed effect model was used for analyses without heterogeneity. Statistical heterogeneity was assessed using the I-squared (I^2^) statistic test [37]. We performed a meta-regression and subgroup analysis to explore possible causes of heterogeneity. To evaluate publication bias, we visually examined the asymmetry of the funnel plot and conducted Egger’s test [38]. The p-value was < 0.05, indicating statistical evidence for publication bias. Additionally, a counter-enhanced funnel plot was employed to differentiate between asymmetry caused by publication bias and asymmetry caused by other factors. A sensitivity analysis was performed to assess the impact of a single study on the overall estimate.

## 3. Results

### 3.1. Search results

**Fig 1:**
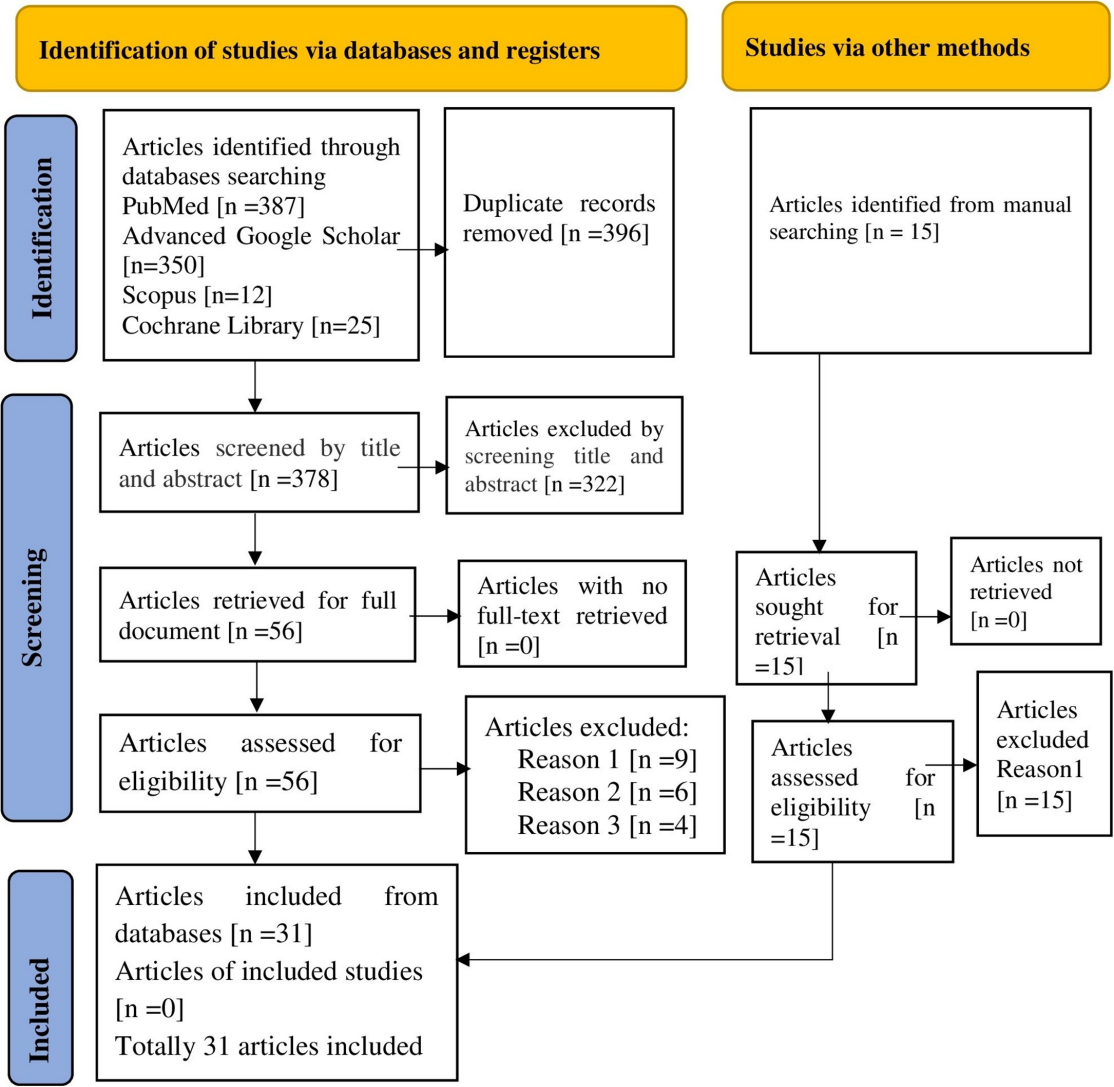
A PRISMA flow diagram showing the screening and inclusion of the studies. Reason 1: Poor methodological quality of data. Reason 2: The full textile article is unavailable. Reason 3: Not reporting the outcome of interest.

Initially, a total of 774 studies were retrieved from electronic databases. After removing 396 duplicate items, 378 articles remained for review based on their title and abstracts. Subsequently, 322 research papers were excluded. Four of the remaining 56 full-text papers were excluded as they did not report the outcome of interest, six were excluded due to the unavailability of full-text articles, and nine were excluded due to low methodological quality. Finally, 31 studies that met the inclusion criteria were selected and invited to participate in the study (Fig 1).

### 3.2. Study characteristics

The analysis incorporated studies published between 2013 and 2023. A comprehensive total of 31 articles were utilized to determine the overall prevalence of hepatitis B virus infection among pregnant women in Africa. The sample size ranged from 124 to 12138; twenty-seven out of the thirty-one studies (87.1%) were facility-based cross-sectional studies, three (9.68%) were retrospective medical record reviews, and one (3.23%) was a prospective cohort study.

The methodological quality score was more significant than or equal to seven in all twenty-one studies. Out of 31 articles, 10 (32.26%) were conducted in Ethiopia, seven (22.58%) in Nigeria, two (6.45%) in Ghana, two (6.45%) in South Sudan, two (6.45%) in Sudan, and one (3.23%) each from Uganda, Burkina Faso, Chad, Somalia, South Africa, Egypt, Cameroon and Gambia (Table 1).

**Table 1:** Characteristics of studies included in the review from 2013 to 2023 (N = 33,967).

Author, Year of Publication	Country	Study Population	Study Type	Sample Size	Pregnant women tested positive	Prevalence [95%CI]	Quality
Mortada EL-Shabrawi et al., 2014 [39]	Egypt	Pregnant Women	Facility-based cross-sectional survey	2,000	35	1.8% [1.7–10.2]	7
Frambo et al., 2014 [40]	Cameroon	Pregnant Women	Facility-based cross-sectional survey	176	17	9.7% [5.7–15]	7
Mohammed Hammad Abuelgasim and Mohammed Basheer Koko Baraka, 2015 [18]	Sudan	Pregnant Women	Facility-based cross-sectional survey	160	12	7.5%[5.4–9.8]	7
Dahie and Heyle, 2017 [8]	Somalia	Pregnant Women	Facility-based cross-sectional survey	364	15	4.1%[3.3–6.4]	7
Stephen Kirbak et al., 2017 [41]	South Sudan	Pregnant Women	Facility-based cross-sectional survey	280	31	11%[8.1–12.8]	8
Atilola G et al., 2018 [42]	Nigeria	Pregnant Women	Facility-based cross-sectional survey	353	37	10.5% [7.5–14.2]	7
Gasim et al., 2019 [43]	Sudan	Pregnant Women	Facility-based cross-sectional survey	900	162	18%[15.3–19.7]	8
Bittaye et al., 2019 [44]	Gambia	Pregnant Women	Facility-based cross-sectional survey	426	39	9.2[6.9–13.2]	9
Peter Asaga et al., 2019 [45]	Nigeria	Pregnant Women	Facility-based cross-sectional survey	200	39	19.5[14.2–21.5]	8
Magaji et al., 2020 [46]	Nigeria	Pregnant Women	Facility-based cross-sectional survey	3238	241	7.4[6.6–8.4]	8
Kwadzokpui et al., 2020 [47]	Ghana	Pregnant Women	Facility-based cross-sectional survey	213	7	3.3[2.3–3.9]	8
Bancha et al., 2020 [16]	Ethiopia	Pregnant Women	Facility-based cross-sectional survey	675	49	7.3%[5–9]	9
Dortey BA et al., 2020 [48]	Ghana	Pregnant Women	Facility-based cross-sectional survey	221	17	7.7%[6.7,8.7]	8
Michael Pou and Dube, 2021 [49]	South Sudan	Pregnant Women	Facility-based cross-sectional survey	234	16	6.8% [3.8–10.3]	9
Tadiwos et al., 2021 [13]	Ethiopia	Pregnant Women	Facility-based cross-sectional survey	479	44	9.2%[4.2–14.2]	8
Atalay et al., 2021 [50]	Ethiopia	Pregnant Women	Facility-based cross-sectional survey	215	11	5.1%[1.1–12.2]	8
Iliyasu et al., 2022 [51]	Nigeria	Pregnant Women	Facility-based cross-sectional survey	394	46	11.7% [6.1–17.1]	7
Kassaw et al., 2022 [52]	Ethiopia	Pregnant Women	Facility-based cross-sectional survey	381	25	6.6%[4.2–8.9]	8
Atwine et al., 2022 [25]	Uganda	Pregnant Women	Facility-based cross-sectional survey	341	7	2.1%[0.5–3.5]	8
Argaw et al., 2022 [9]	Ethiopia	Pregnant Women	Facility-based cross-sectional survey	338	11	3.3%[1.5–5]	7
Amaike C et al., 2022 [27]	Nigeria	Pregnant Women	Retrospective chart review	706	82	11.6%[6.5–18.1]	7
Afolabi A et al., 2022 [53]	Nigeria	Pregnant Women	Retrospective chart review	4300	110	2.6%[1.8–3.2]	7
Gebretsadik., et al., 2022 [54]	Ethiopia	Pregnant Women	Facility-based cross-sectional survey	124	10	8.1%[9.8–13.8]	7
Joseph Davey et al., 2022 [55]	South Africa	Pregnant Women	Retrospective chart review	1194	8	6.7%[3.4–13.2]	8
Ukpe et al., 2023 [56]	Nigeria	Pregnant Women	Facility-based cross-sectional survey	291	23	7.9%[4.8–11.2]	8
Umer et al., 2023 [12]	Ethiopia	Pregnant Women	Facility-based cross-sectional survey	300	24	8%[5.3–11]	7
Kampe et al., 2023 [11]	Ethiopia	Pregnant Women	Facility-based cross-sectional survey	368	21	5.7%[3.7–8.6]	7
Ouoba et al., 2023 [57]	Burkina Faso	Pregnant Women	Facility-based cross-sectional survey	1622	106	6.5%[5.4–7.8]	10
Israel E et al., 2023 [23]	Ethiopia	Pregnant Women	Facility-based cross-sectional survey	484	8	1.7%[1.5–4.1]	9
Debsikreo N et al., 2023 [7]	Chad	Pregnant Women	Facility-based cross-sectional survey	458	33	7.2%[5–10]	7
Tesfu et al., 2023 [14]	Ethiopia	Pregnant Women	prospective cohort	12138	369	3.1%[1.8–4.9]	8
Total				33,967	1701	7.5%[5.1–10.6]	7.8

Where; HBV: hepatitis B Virus; N: Sample Size

### 3.3. Pooled prevalence of hepatitis B virus among pregnant women in Africa

**Fig 2:**
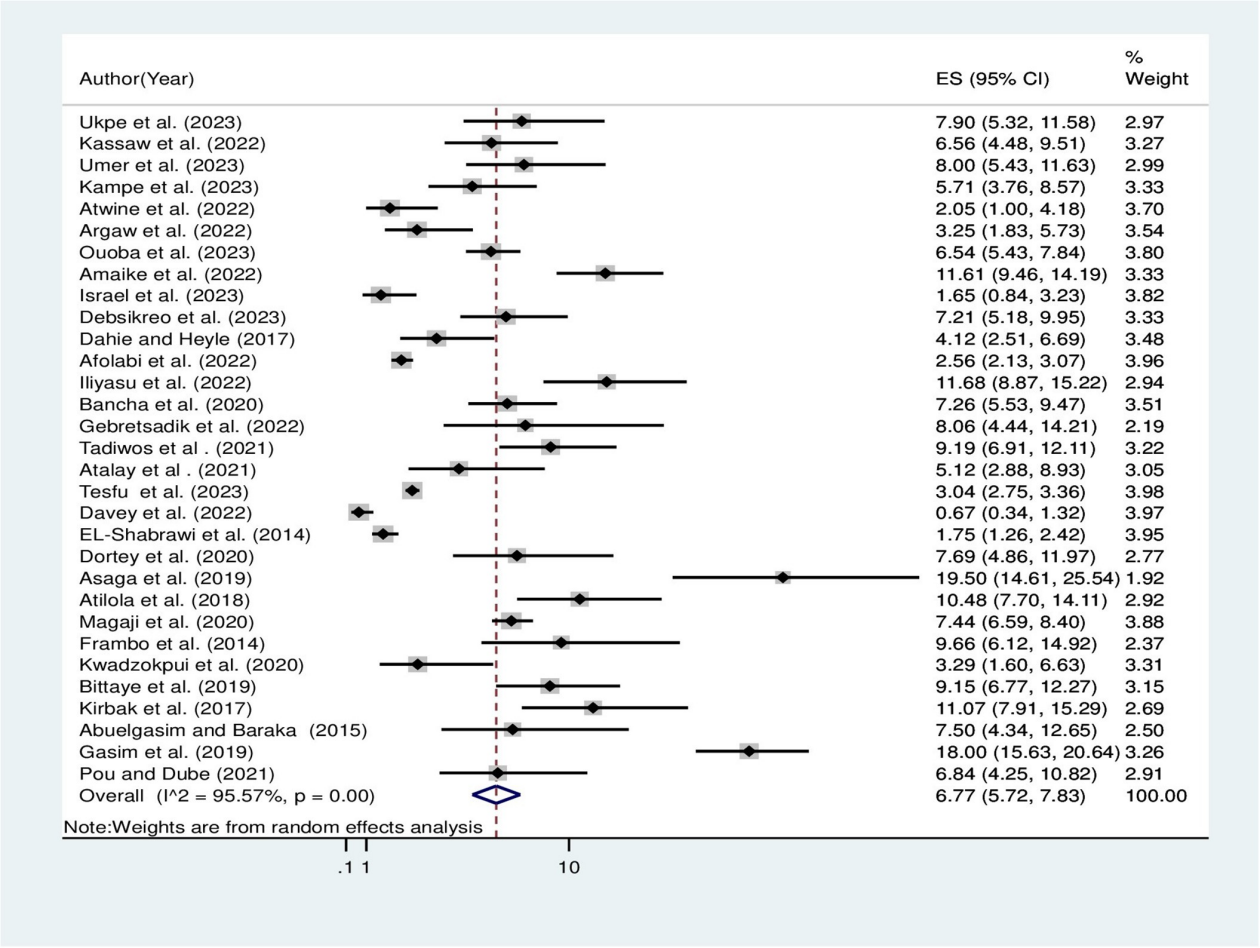
Overall pooled prevalence of hepatitis B virus among pregnant women in Africa, 2023.

The overall pooled prevalence of hepatitis B virus among pregnant women in Africa using the fixed effect model was 3.16% [95% CI: 2.98, 3.35]. When using the fixed effect model, the pooled effect size of the hepatitis B virus among pregnant women showed significant heterogeneity among the included studies (I^2^) of 95.57% (P = 0.00). As a result, the final prevalence was determined using a random-effect model to account for the observed heterogeneity. The pooled prevalence of hepatitis B virus among pregnant women in Africa using the random effect model was 6.77% [95% CI: 5.72, 7.83] (Fig 2).

### 3.4. Subgroup analysis by country and region

Subgroup analysis revealed that the aggregated estimate of HBV infection among pregnant women was assessed based on country and region. Consequently, we identified variations in the prevalence of HBV infection among the countries reviewed. The specific prevalence rates of HBV were 9.73%, 5.44%, 2.05%, 6.54%, 7.21%, 4.12%, 0.67%, 1.75%, 4.68%, 9.66%, 9.15%, 8.69%, and 15.12% in Nigeria, Ethiopia, Uganda, Burkina Faso, Chad, Somalia, South Africa, Egypt, Ghana, Cameroon, Gambia, South Sudan, and Sudan, respectively. Additionally, regional disparities in the prevalence of HBV infection were also observed in this review. The detailed prevalence rates of HBV were 8.58%, 4.94%, 7.21%, 0.67%, and 9% in the West, East, Central, Southern, and Northern regions of Africa, respectively (Table 2).

**Table 2:** Subgroup analysis of the pooled prevalence of hepatitis B virus among pregnant women in Africa, 2023.

Variables	Characteristics	NS	Pooled prevalence 95%CI	I^2^	P-value
Country	Nigeria	7	9.73[6.32,13.14]	97.08%	0.00
	Ethiopia	10	5.44[3.95,6.93]	87.37%	0.00
	Uganda	1	2.05[1,4.18]	-	-
	Burkina Faso	1	6.54[5.43,7.84]	-	-
	Chad	1	7.21[5.18,9.95]	-	-
	Somalia	1	4.12[2.51,6.69]	-	-
	South Africa	1	0.67[0.34,1.32]	-	-
	Egypt	1	1.75[1.26,2.42]	-	-
	Ghana	2	4.68[2.71,6.66]	-	-
	Cameroon	1	9.66[6.12,14.92]	-	-
	Gambia	1	9.15[6.77,12.27]	-	-
	South Sudan	2	8.69[6.26,11.11]	-	-
	Sudan	2	15.12[12.98,17.26]	-	-
Region	West	12	8.58[6.33,10.83]	95.57%	0.00
	East	12	4.94[3.71,6.17]	85.22%	0.00
	Central	1	7.21[5.18,9.95]	-	-
	Southern	1	0.67[0.34,1.32]	-	-
	Northern	5	9[1.8,16.21]	97.83	0.00

Where: NS: is the number of studies; CI: confidence Interval, and I^2^: I–Squared.

Meta-regression analysis revealed that the observed heterogeneity could be accounted for by two factors: publication year (P = 0.002) and sample size (P = 0.004) (Table 3). It is widely recognized that meta-analyses focusing on prevalence often exhibit substantial variability. Consequently, genuine heterogeneity in prevalence estimates is expected to arise due to variations in the timing and location of the conducted studies. These factors should be interpreted cautiously as they may not be discriminatory [58]. I^2^ estimates can sometimes be unreliable due to inadequate power and precision. Factors contributing to high heterogeneity include time-dependent bias, dependence on sample size, study location, regional differences, mean/median sample size, HBV screening methods, methodological quality, and individual variation among study participants [58, 59].

**Table 3:** Meta-regression analysis of factors influencing between-study heterogeneity for the pooled prevalence of HBV infection among pregnant women in Africa, 2023.

Source of heterogeneity	Coef	Std. Err	T	P>|t|	[95% Conf. Interval]
Year of publication	.5583528	.1608809	3.47	0.002	.2293144,.8873912
Sample size	.94912222	.3054847	3.11	0.004	.3243358,1.573909

### 3.5. Publication bias

**Fig 3:**
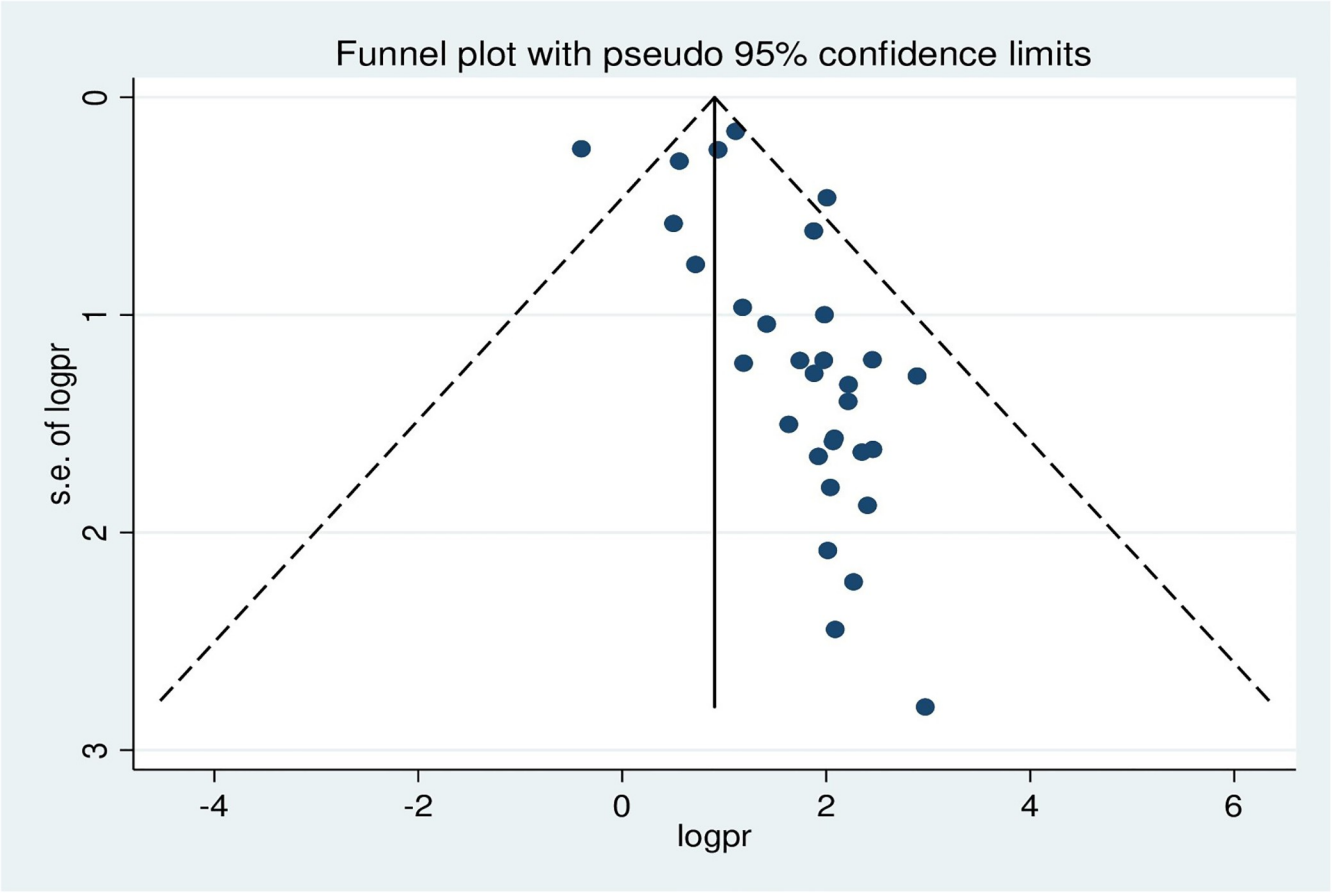
A funnel plot illustrates publication bias regarding the prevalence of hepatitis B virus among pregnant women in Africa, 2023.

Publication bias was assessed using a funnel plot and Egger’s regression test. The funnel plot depicted evidence of asymmetry (Fig 3).

**Fig 4:**
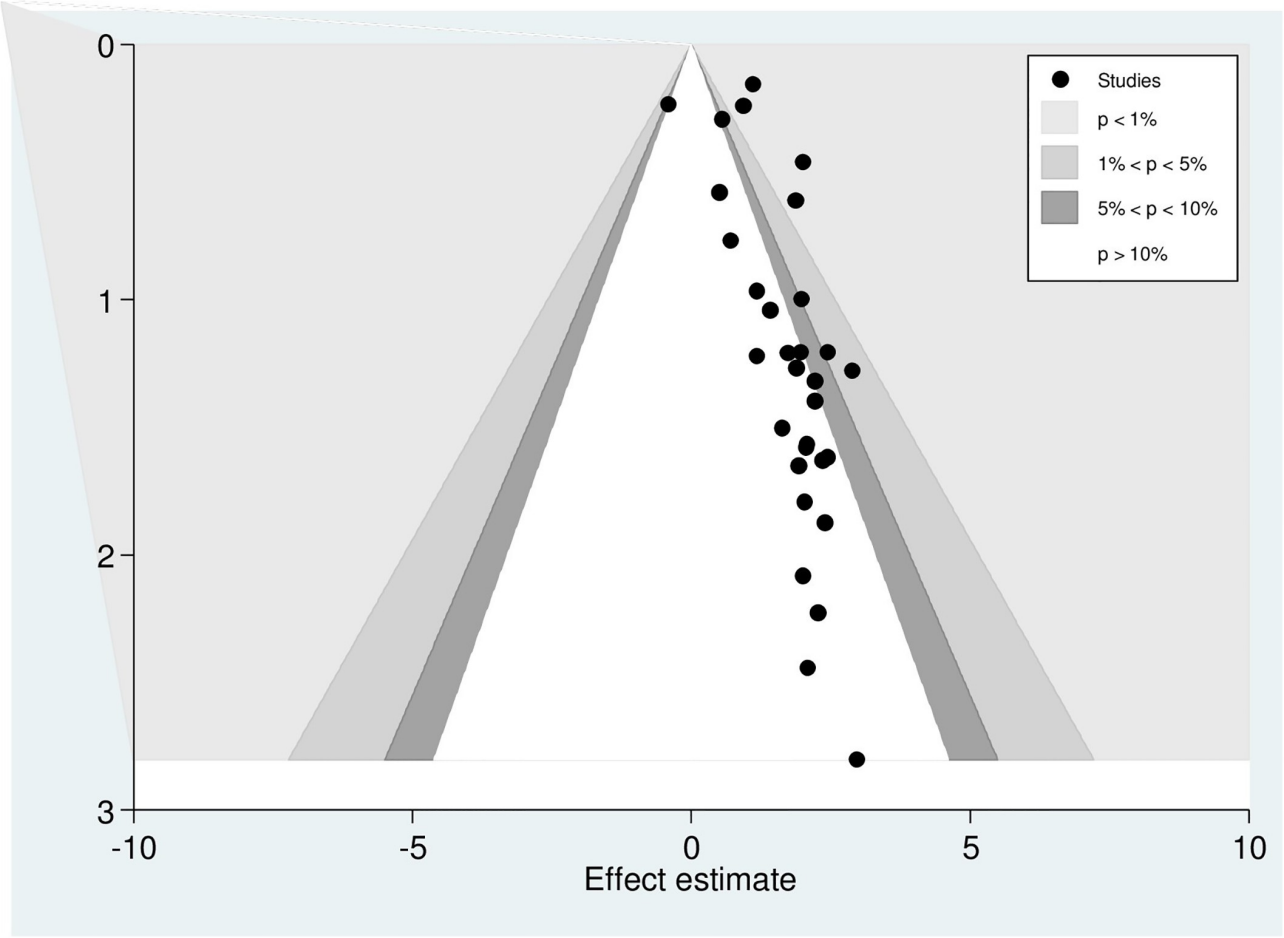
Counter-enhanced funnel plots for publication bias for the prevalence of hepatitis B virus among pregnant women in Africa, 2023.

Egger’s regression test was statistically significant, with a P-value of 0.00. However, it is essential to note that the funnel plot and Egger’s test primarily assess the risk of slight study bias, and smaller studies tend to have more significant variance. While an asymmetrical plot could indicate publication bias, other factors could explain funnel plot asymmetry. To further investigate this, we conducted a contour-enhanced funnel plot to differentiate between asymmetry caused by publication bias and asymmetry caused by other factors. The contour-enhanced funnel plot suggests that the "missing" studies are expected to be located in areas of high statistical significance (shaded areas), while most available studies are not statistically significant. This indicates that the observed asymmetry may not be solely due to publication bias based on statistical significance. Therefore, it is probable that the asymmetry can be attributed to several other factors, including but not limited to study size, study effect, study design, study location, regional variances, HBV screening methods, methodological quality, and individual variations among study participants (Fig 4).

**Fig 5:**
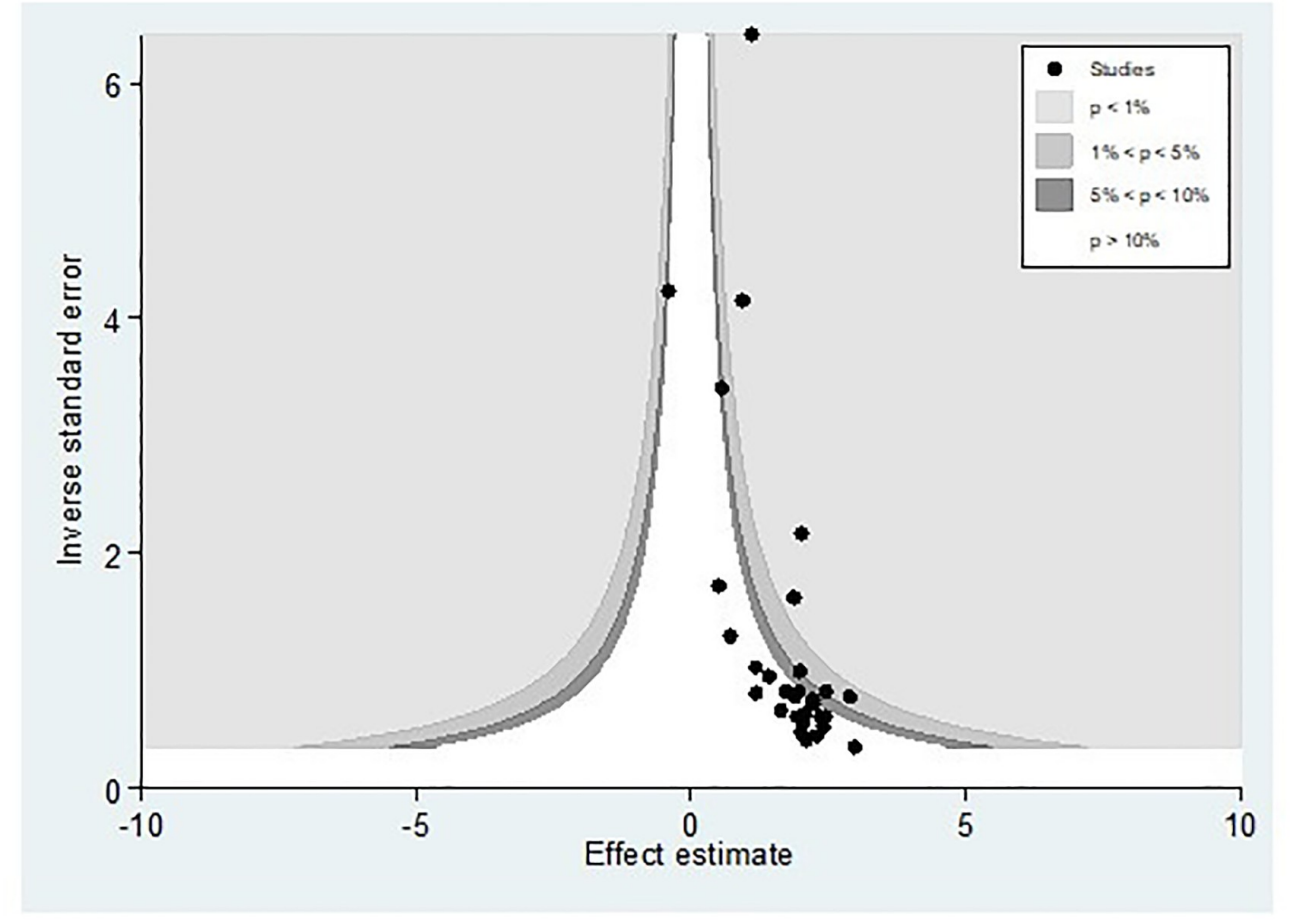
Metric inverse counter-enhanced funnel plots of publication bias for the prevalence of hepatitis B virus among pregnant women in Africa, 2023.

Similar findings were observed when conducting a metric (inverse) counter-enhanced funnel plot (Fig 5).

### 3.6. Sensitivity analysis

**Fig 6:**
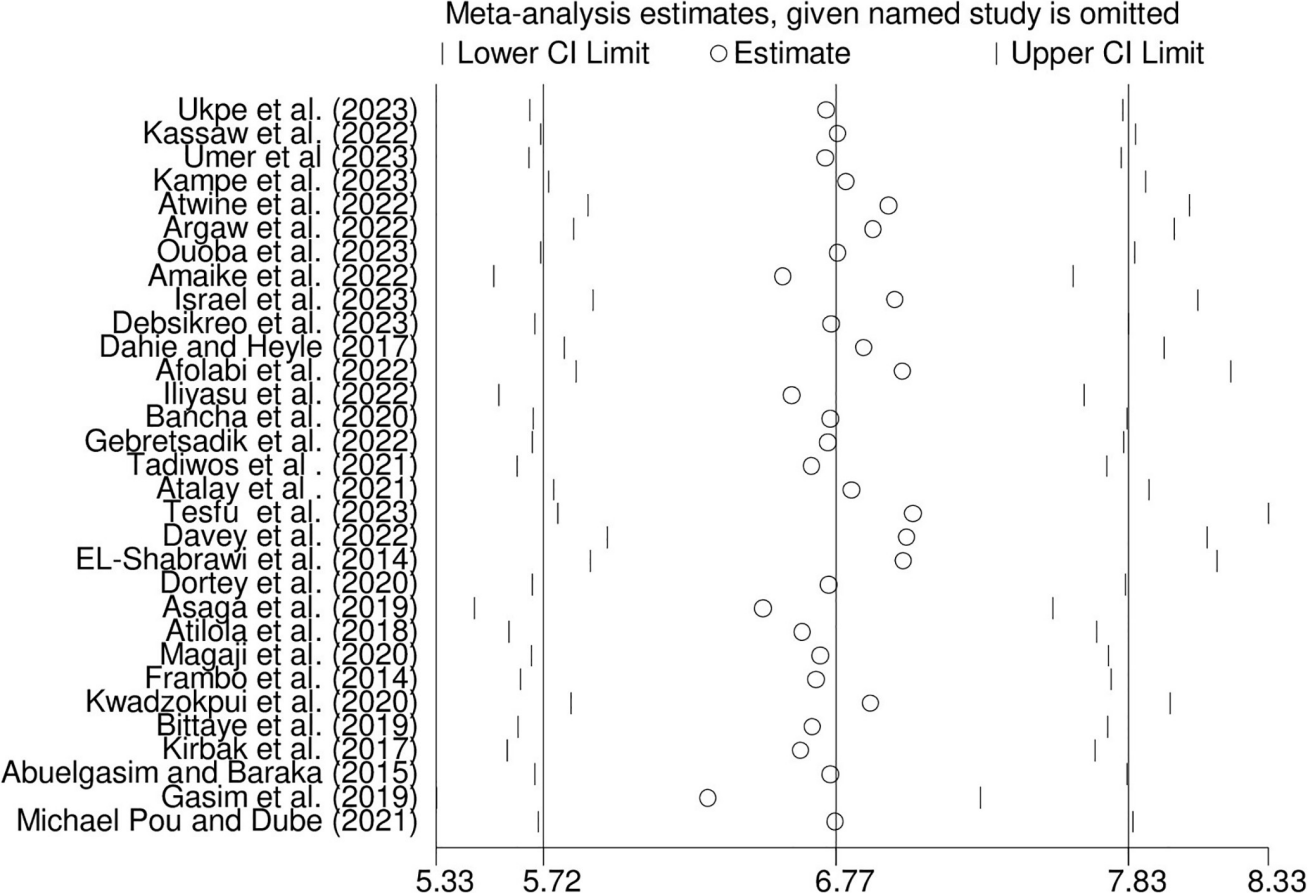
Sensitivity analysis for the prevalence of hepatitis B virus among pregnant women in Africa, 2023.

We performed a sensitivity analysis to assess how each study affected the overall summary estimate of the meta-analysis. No studies were outside the confidence bounds in the sensitivity analysis, suggesting that all studies had a nearly equal influence on the pooled prevalence (Fig 6).

## 4. Discussion

Among the various viruses that cause hepatitis, HBV is responsible for both acute and chronic infections. It is the most prevalent and severe liver infection globally, leading to significant morbidity and mortality [8, 9, 60]. However, there is limited documentation regarding the national prevalence of hepatitis B infection among pregnant women. The concern surrounding HBV infection in pregnant women stems from the potential transmission of the virus to their new-borns during delivery. By identifying the hepatitis status of pregnant mothers, the risk of virus transmission can be mitigated, thereby reducing the likelihood of chronic hepatitis development in infants.

To contribute to the expanding body of evidence on HBV in Africa, we have conducted a systematic review and meta-analysis to investigate the prevalence of HBV among pregnant women. Our analysis encompasses studies published between July 2013 and July 2023. In this systematic review and meta-analysis, 31 studies indicated a relatively high prevalence rate of 6.77%. According to the classification of HBV endemicity based on HBsAg prevalence–low (<2%), lower-intermediate (2–4.99%), higher-intermediate (5–7.99%), and high (>8%) [32, 61], our findings suggest a higher-intermediate endemicity of HBV infection among pregnant women in Africa. The reported range of HBV infection among pregnant women in Africa varies from 9% to 20% [8]. These results similar with a meta-analysis conducted in Nigeria (6.49%) [32], Ethiopia (5.78%) [60] and (7.4%) [62], a cross-sectional study conducted in Khartoum, Sudan(7.5%) [18], a retrospective review of pregnant women in Ghana (6%) [17], Wolaita Sodo in southern Ethiopia (7.5%) [63], Deder Hospital in Eastern Ethiopia (6.9%) [64], and Addis Ababa, Ethiopia (6%) [65]. However, our findings are lower than the prevalence rates reported in a meta-analysis study in Nigeria (9.5%) [22], Kenya (9.3%) [19], Cameroon (11.2%) [66], a cross-sectional study in Nigeria (10.9%) [67], Northwest Ethiopia (8%) [5], and Tigray in Ethiopia (11.6%) [68]. On the other hand, our meta-analysis study reveals higher prevalence compared to a systematic review and meta-analysis conducted in Ethiopia (4.75%) [69], Bangladesh (4%) [28], Ghana (4.6%) [10], Ambo town in central Ethiopia (4.99%) [70], Arba Minch South Ethiopia (4.3%) [71], Eritrea (3.2%) [20], Iranian pregnant women (1.2%) [72] and a cross-sectional study conducted in Eretria(3.2%) [20], (3.1%) in Rwanda [21] and (1.3%) Cairo, Egypt [2].

In the subgroup analysis, there was a significant variation in the prevalence of HBV infection among the subnational regions of Africa. According to the findings of this analysis, the northern region showed the highest prevalence of HBV at 9%, while the eastern region had the lowest prevalence at 4.94%. This discrepancy can be attributed to variations in the number of studies included in the analysis, as the northern region of Africa was limited to only five.

The explanation for the overall variations in the prevalence of HBV infection among pregnant women can be attributed to a combination of factors, including sociodemographic characteristics, cultural and environmental differences, behavioural risk factors for HBV infection, methodological variances, and natural disparities associated with different geographical situations. Factors such as awareness levels, sample size, study participants, study design, and infection prevention practices within different communities across countries and regions also contribute to the observed differences.

The findings of this study provide essential data for assessing the effectiveness of existing prevention and control strategies in Africa. Moreover, this study serves as a valuable resource for developing and implementing efficient public health management strategies, with the ultimate goal of achieving elimination by 2030. Prevention of mother-to-child transmission (PMTCT) of HBV infection is a crucial component of initiatives aimed at advancing the elimination of viral hepatitis. According to existing guidelines, it is advised to conduct maternal screening, administer antiviral therapy during the third trimester of high-risk pregnancies, implement universal and timely HBV birth dose vaccination, and administer post-exposure prophylaxis with hepatitis B immunoglobulin to selected neonates [73–76].

## 5. Limitation of the study

Significant variation was observed among the included studies, which may affect the reliability of the combined estimate. A thorough analysis was conducted to examine the potential sources of this variation. The findings indicated that differences in screening methods, study settings, and countries could have influenced the observed variation. The high levels of variation pose challenges for similar meta-analytic studies on HBV in Africa. Additionally, there was variability in the reporting quality of the studies included. However, it is vital to acknowledge the possibility of underestimating or overestimating the outcome variable. Obtaining accurate data on the prevalence of HBV infection among pregnant women is crucial for effectively managing the vertical transmission of the disease. A search was conducted in four databases, and only studies published in English were included. This may have resulted in the exclusion of relevant articles and specific reports on the prevalence of HBV infection among pregnant women in Africa.

## 6. Conclusion

The pooled prevalence of hepatitis B infection among pregnant women in Africa was found to be intermediate-high.

## 7. Recommendation

Therefore, governmental and non-governmental organizations must augment their endeavours towards integrating reproductive health services, specifically HBV screening, as standard elements of ANC clinics in Africa. Moreover, it is crucial to deliver comprehensive health education and treatment when a positive HBV status is identified in order to avert HBV transmission from mother to child. Consequently, policymakers in the maternal health sector are strongly advised to enforce HBV immunization programs tailored specifically for pregnant women and their new-born infants who have been exposed to the virus. Furthermore, researchers should undertake analogous studies on related factors.

## Supporting information

S1 File Search terms summery. (DOCX)

S2 File Data abstraction and quality assessment form. (DOCX)

S1 Checklist PRISMA 2020 checklist. (DOCX)
