# Supplementary material for: Prevalence of hepatitis B virus infection among pregnant women in Africa: A systematic review and meta-analysis
Source: PLoS One. 2024 Jul 16;19(7):e0305838. doi: 10.1371/journal.pone.0305838 (PMC11251621; doi:10.1371/journal.pone.0305838)
Supplement: S1 File — (DOCX) [file pone.0305838.s001.docx]

**Additional file 1: Search terms summery**

| Database | Search Terms |
| --- | --- |
| PubMed | ((("Prevalence"[Mesh] OR "Epidemiology"[Mesh] OR "Cross-Sectional Studies"[Mesh]) AND ("Hepatitis B"[Mesh] OR "Hepatitis B, Chronic"[Mesh])) AND ("Pregnant Women"[Mesh])) AND ("Africa"[Mesh]). |
| Advanced Google Scholar | "Prevalence “AND "Hepatitis B Virus “AND "Infection" AND "Pregnant Women" AND "Africa". |
