# Supplementary material for: Prevalence of hepatitis B virus infection among pregnant women in Africa: A systematic review and meta-analysis
Source: PLoS One. 2024 Jul 16;19(7):e0305838. doi: 10.1371/journal.pone.0305838 (PMC11251621; doi:10.1371/journal.pone.0305838)
Supplement: S2 File — (DOCX) [file pone.0305838.s002.docx]

**I: DATA ABSTRACTION AND QUALITY ASSESSMENT FORM**

Reviewer: _______________________________________________.

Date: ___________________________________________________.

Title of article: ___________________________________________.

1. First author’s name:
2. Year of publication:
3. Year of study:
4. Type of study:
5. Region of study:
6. Sub-region of study:
7. Location of study (state):
8. Sample size:
9. Tests conducted :
10. Method for each test:
11. Number screened for HBsAg:
12. Number positive for HBsAg:
13. Number negative for HBsAg:
14. Number screened for HBeAg:
15. Number positive for HBeAg:
16. Number negative for HBeAg:

**II: Critical Appraisal Checklist**

**1:** **For cross-sectional study**

| S/N | **Question** | **Selection** | **Comparability** | **Outcome** |
| --- | --- | --- | --- | --- |
| 1 | Representativeness of the sample | 1. Truly representative of the average in the target population(all subjects or random sampling) 2. Somewhat representative of the average in the target group (non-random sampling) 3. Selected group of users/convenience sample. 4. No description of the derivation of the included subjects. | - | - |
| 2 | Sample size | 1. Justified and satisfactory (including sample size calculation). 2. Not justified. 3. No information provided | - | - |
| 3 | Non-respondents: | 1. Proportion of target sample recruited attains pre-specified target or basic summary of non-respondent characteristics in sampling frame recorded. 2. Unsatisfactory recruitment rate, no summary data on non-respondents. 3. No information provided | - | - |
| 4 | Ascertainment of the exposure (risk factor) | 1. Vaccine records/vaccine registry/clinic registers/hospital records only. 2. Parental or personal recall and vaccine/hospital records. 3. Parental/personal recall only. | - | - |
| 5 | Comparability of subjects in different outcome groups on the basis of design or analysis. Confounding factors controlled. | - | 1. Data/ results adjusted for relevant predictors/risk factors/confounders e.g. age, sex, time since vaccination, etc.  2. Data/results not adjusted for all relevant confounders/risk factors/information not provided. | - |
| 6 | Assessment of outcome | - | - | 1. Independent blind assessment using objective validated laboratory methods.  2. Unblinded assessment using objective validated laboratory methods.  3. Used non-standard or non-validated laboratory methods with gold standard.  No description/non-standard laboratory methods used. |
| 7 | Statistical test | - | - | 1. Statistical test used to analyses the data clearly described, appropriate and measures of association presented including confidence intervals and probability level (p value).  2. Statistical test not appropriate, not described or incomplete. |
| **II:For case control study** | | | | |
| 1 | Is the case definition adequate? | 1. yes, with independent validation 2. yes, eg record linkage or based on self-reports 3. no description | - | - |
| 2 | Representativeness of the cases | 1. consecutive or obviously representative series of cases 2. potential for selection biases or not stated | - | - |
| 3 | Selection of Controls | 1. community controls 2. hospital controls 3. no description | - | - |
| 4 | Definition of Controls | 1. no history of disease (endpoint) 2. no description of source | - | - |
| 5 | Comparability of cases and controls on the basis of the design or analysis | - | 1. Study controls for _______(Select the most important factor.)  2. Study controls for any additional factor (This criteria could be modified to indicate specific control for a second important factor.) | - |
| 6 | Ascertainment of exposure | - | - | 1. secure record (eg surgical records)  2.structured interview where blind to case/control status  3. interview not blinded to case/control status d)  4. written self-report or medical record only  5. no description |
| 7 | Same method of ascertainment for cases and controls | - | - | 1.yes  2.no |
| 8 | Non-Response rate | - | - | 1. same rate for both groups  2. non respondents described  3. rate different and no designation |
| **III: Cohort study** | | | | |
| 1 | Representativeness of the exposed cohort | 1. truly representative of the average _______________ (describe) in the community 2. somewhat representative of the average ______________ in the community 3. selected group of users eg nurses, volunteers 4. no description of the derivation of the cohort | - | - |
| 2 | Selection of the non-exposed cohort | 1. drawn from the same community as the exposed cohort 2. drawn from a different source 3. no description of the derivation of the non-exposed cohort | - | - |
| 3 | Ascertainment of exposure | 1. secure record (eg surgical records) 2. structured interview 3. written self-report 4. no description | - | - |
| 4 | Demonstration that outcome of interest was not present at start of study | 1.Yes  2.No | - | - |
| 5 | Comparability of cohorts on the basis of the design or analysis | - | 1. Study controls for ________(select the most important factor)  2. Study controls for any additional factor(this criteria could be modified to indicate specific control for a second important factor) | - |
| 6 | Assessment of outcome | - | - | 1. independent blind assessment  2. record linkage  3. self-report  3. no description |
| 7 | Was follow-up long enough for outcomes to occur | - | - | 1. yes (select an adequate follow up period for outcome of interest) 2. no |
| 8 | Adequacy of follow up of cohorts |  |  | 1. complete follow up - all subjects accounted for  2. subjects lost to follow up unlikely to introduce bias - small number lost - > ____ % (select an adequate %) follow up, or description provided of those lost)  3. follow up rate < ____% (select an adequate %) and no description of those lost  4. no statement |
